# Characterisation of Enterocins Produced by Antilisterial *Enterococcus faecium* BH04, BH12, BH84, and BH99 and In Vitro/In Situ Inhibition of *Listeria monocytogenes*

**DOI:** 10.1002/fsn3.70142

**Published:** 2025-04-01

**Authors:** Haktan Aktaş

**Affiliations:** ^1^ Department of Food Engineering, Faculty of Agriculture Ataturk University Erzurum Turkey

**Keywords:** enterocin, *Enterococcus faecium*, in vitro, *Listeria monocytogenes*, milk model

## Abstract

*Listeria monocytogenes* is a food pathogen that can cause fatal infections, especially in newborns, elderly individuals, and immunocompromised individuals. In recent years, research on novel antibiotics and natural antimicrobial agents of plant or microbial origin has become increasingly important in the face of multiple antibiotic resistance. In this study, enterocins produced by *Enterococcus faecium* BH04, BH12, BH84, and BH99 were investigated in vitro and in situ as antilisterial agents. The results showed that all strains had bacteriocin activity of 0.4 kAU/mL (400 AU/mL) after 3 h and 25.6 kAU/mL (25600 AU/mL) after 6 h in M17 broth. The strains inhibited the growth of *Listeria monocytogenes* ATCC 7644 in BHI. Furthermore, *E. faecium* BH04, BH12, BH84, and BH99 had bacteriostatic potential, whereas enterocins BH04, BH12, BH84, and BH99 had bactericidal potential in a milk model (in situ). The polymerase chain reaction results revealed that all strains had *entA*, *entB*, and *entP* genes encoding enterocin but not the *entL50* gene. The enterocins BH04, BH12, BH84, and BH99 retained their antilisterial activity up to 100°C (10 min), up to pH 10, and against catalase, lysozyme, and all chemicals used in this study. On the other hand, ultraviolet light reduced the antilisterial activity of enterocin BH12 by 75% and that of enterocin BH04, BH84, and BH99 by 50%. It was concluded that *E. faecium* BH04, BH12, BH84, and BH99 could be used as a co‐culture in fermented products and that enterocins produced by the strains could be used as antilisterial agents.

## Graphical abstract

In this study, enterocins produced by *Enterococcus faecium* BH04, BH12, BH84, and BH99 were investigated in vitro and in situ as an antilisterial agent. All strains had bacteriocin activity of 0.4 kAU/mL at 3 h and 25.6 kAU/mL at 6 h and inhibited the growth of *Listeria monocytogenes* ATCC 7644 in BHI.

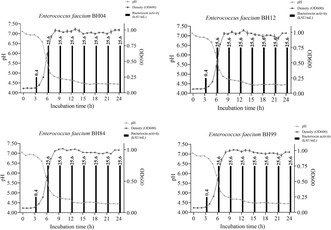

## 1 Introduction

Foodborne pathogens such as *Listeria monocytogenes* ( *L. monocytogenes* ) have the potential to threaten food safety and adversely affect human health by causing many diseases (FAO/WHO 2021). *L. monocytogenes* , one of the most important foodborne pathogens, can cause some serious diseases, including septicaemia, meningitis, and foetal loss (Tauxe 2002). While the mortality rate of listeriosis was 25% at the beginning of the twenty‐first century (Tauxe 2002), recent reports unfortunately indicate that this rate has not decreased (Matsuda et al. 2023). This situation demonstrates the need for effective antibiotics and/or natural antimicrobials (Church and McKillip 2021). Furthermore, as antibiotic‐resistant bacterial infections are thought to cause too many deaths each year (e.g., 23,000 deaths in the United States alone), natural antimicrobial agents such as bacteriocins and plant extracts have attracted considerable interest in recent years to combat pathogens (Chen et al. 2024). Bacteriocins, such as enterocin, lactacin, brevicin, caseicin, lacticin, and reutericin, can be produced by many lactic acid bacteria (LAB) (Klaenhammer 1993) and offer important food safety advantages because they are safe, nontoxic to humans, generally heat and pH stable, and have a broad antimicrobial spectrum including many pathogens (Gálvez et al. 2007). For these reasons, it is essential to study and characterize antilisterial bacteriocins produced by LAB, especially to reduce the virulence potential of *L. monocytogenes* .

This study aimed to characterize the enterocins produced by antilisterial *Enterococcus faecium* ( *E. faecium* ) BH04, BH12, BH84, and BH99 in terms of several aspects, including antibacterial spectrum, detection of enterocin genes, sequencing, and determination of residual activity against many treatments such as temperature, pH, enzyme, chemical, and ultraviolet (UV) light. In addition, the in vitro and in situ inhibitory effects of the enterocins on *L. monocytogenes* ATCC 7644 were investigated.

## 2 Materials and Methods

### 2.1 Strains and Growth Conditions

In this study, the bacteriocinogenic *E. faecium* BH04, BH12, BH84, and BH99, which were isolated from raw cow's milk and were determined to have bacteriocin‐induced antimicrobial activity in a previous study by Çetin and Aktaş (2024), were used as enterocin‐producing strains. The 16S rRNA identification, probiotic characterization, and safety assessment results of these isolates are presented in Table S1. The incubation conditions of the strains in this study were on M17 agar or broth (Merck, Darmstadt, Germany) at 37°C for 24 h. In addition, all other isolates used in this study and their growth conditions are listed in Table 1.

**TABLE 1:** Antibacterial spectrum of the enterocins produced by the *Enterococcus* spp. on some pathogens and nonpathogen bacteria^a^.

Isolates	Source	Growth conditions	*Enterococcus* strains (inhibition zone diameter, mm)			
			*Enterococcus faecium* BH04	*Enterococcus faecium* BH12	*Enterococcus faecium* BH84	*Enterococcus faecium* BH99
*Enterococcus faecium* BH04	Çetin and Aktaş (2024)	M17, 37°C	ND	—	—	—
*Enterococcus faecium* BH12	Çetin and Aktaş (2024)	M17, 37°C	—	ND	—	—
*Enterococcus faecium* BH84	Çetin and Aktaş (2024)	M17, 37°C	—	—	ND	—
*Enterococcus faecium* BH99	Çetin and Aktaş (2024)	M17, 37°C	—	—	—	ND
*Listeria monocytogenes*	ATCC 7644	BHIA, 37°C	+ (20.5 ± 0.7)	+ (20.3 ± 0.4)	+ (19.3 ± 0.4)	+ (19.8 ± 0.4)
*Staphylococccus aureus*	ATCC 29213	NA, 37°C	—	—	—	—
Methicillin‐resistant *Staphylococccus aureus*	ATCC 43300	NA, 37°C	—	—	—	—
*Escherichia coli*	ATCC 25922	NA, 37°C	—	—	—	—
*Salmonella Typhimurium*	RSHMB 95091	NA, 37°C	—	—	—	—
*Bacillus spizizenii* (formerly *Bacillus subtilis* subsp. *spizizenii*)	ATCC 6633	BHIA, 30°C	—	—	—	—
*Bacillus cereus*	ATCC 33019	NA, 30°C	—	—	—	—
*Heyndrickxia coagulans* (formerly *Bacillus coagulans* )	ATCC 7050	NA, 30°C	+ (12.5 ± 0.7)	+ (10.0 ± 0.0)	+ (11.5 ± 0.7)	+ (12.3 ± 0.4)
*Streptococcus mutans*	ATCC 25175	BHIA, 37°C	—	—	—	—
*Enterococcus faecalis*	ATCC 29212	M17, 37°C	—	—	—	—
*Streptococcus thermophilus* 212S	Aktaş and Çetin (2024)	M17, 37°C	—	—	—	—
*Lactobacillus delbrueckii* subsp. *bulgaricus* 239 M	Aktaş (2023)	MRS, 37°C	—	—	+ (21.0 + 1.4)	+ (21.0 + 1.4)
*Lactobacillus helveticus*	ATCC 15009	MRS, 37°C	—	—	—	—
*Lactobacillus casei*	ATCC 393	MRS, 37°C	—	—	—	—

Abbreviations: −, negative; +, positive; ATCC, American type culture collection; BHIA, brain heart infusion agar; MRS, De Man, Rogosa, Sharpe agar; NA, nutrient agar; ND, not determined; RSHMB, Refik Saydam Hygiene Centre.

^a^ All results were expressed as mean ± standard deviation. Inhibition zone diameters were expressed as millimeter (mm).

### 2.2 Obtaining Enterocins by Antilisterial *Enterococcus* Spp.

The strains including *E. faecium* BH04, BH12, BH84, and BH99, stored at −80°C in 40% glycerol solution (Merck), were plated on M17 agar (Merck) and incubated at 37°C for 24 h for revival. The fresh cultures were then suspended in phosphate‐buffered saline (PBS, pH 7.2, Merck) at a concentration of 10^8^ cfu/mL. The suspension was added (2%, v/v) to M17 broth (Merck) containing 0.5% glucose (Merck) and incubated at 37°C for 15–18 h. Then, the pH of the broth was adjusted to 6.5–7.0 with 1 N NaOH (Merck) to eliminate acidity, and the mixture was centrifuged at 8000 × *g* for 10 min at 4°C using an Allegra X‐30R Centrifuge (Beckman Coulter Inc., Brea, California, USA). The cell‐free supernatant (CFS) containing enterocin was filtered through a 0.22 μm pore size cellulose acetate filter (Merck) and the catalase enzyme (Sigma‐Aldrich, St. Louis, Missouri, USA) was added (5 mg/mL) to the CFS to eliminate the antimicrobial activity of H_2_O_2_ (Sigma‐Aldrich) (Ogunbanwo et al. 2003).

### 2.3 Microbial Growth Curve and Bacteriocin Activity Against *Listeria monocytogenes* ATCC 7644

M17 broth (Merck) containing 0.5% glucose (Merck) was used to create a microbial growth curve and to determine bacteriocin activity (against *L. monocytogenes* ATCC 7644) of *E. faecium* BH04, BH12, BH84, and BH99 strains. Briefly, an overnight culture was suspended in PBS (Merck) at a concentration of 10^8^ cfu/mL, and the suspension (2%) was added to M17 broth (Merck) containing 0.5% glucose (Merck). After pre‐incubation at 37°C for 18–24 h, the broth (2%) was inoculated again into M17 broth (Merck) containing 0.5% glucose (Merck). The broths were incubated at 37°C for 24 h, and the pH and optical density (OD_600_) of the broths were determined every hour to generate a microbial growth curve. In addition, CFS were harvested from M17 broth (Merck) every 3 h of incubation. The CFS were then serially diluted (two‐fold) with PBS (Merck) and 10 μL of each dilution was spotted onto Brain Heart Infusion Agar (BHIA, Sigma‐Aldrich) inoculated with *L. monocytogenes* ATCC 7644 using a sterile cotton swab. Using the last dilution that inhibited the growth of *L. monocytogenes* ATCC 7644, the enterocin activities of the strains were determined using the formula (two‐fold serial dilution method): Bacteriocin activity (AU/mL) = 2^n^ × 100, where n represents the last dilution that inhibited the growth of *L. monocytogenes* ATCC 7644 (Todorov and Dicks 2007; Murua et al. 2013).

### 2.4 Antibacterial Spectrum of Enterocins

The antibacterial spectrum of the enterocins produced by the *Enterococcus* spp. was determined using the well diffusion method on Gram‐positive and Gram‐negative bacteria listed in Table 1. Briefly, overnight cultures in Table 1 were suspended in PBS (Merck) at a concentration of 10^8^ cfu/mL and spread over the entire agar surface using a sterile cotton swab. Holes of 6 mm diameter were then punched aseptically using a sterile cork borer. One hundred microliters of the CFS were inoculated into the wells. The plates were incubated under the appropriate conditions listed in Table 1. At the end of the incubation, the diameter of the zone around the well was determined and expressed in millimeters (mm) (Balouiri et al. 2016).

### 2.5 In Vitro (Mode of Action) and In Situ (Milk Model) Inhibition of *Listeria monocytogenes*

To determine in vitro inhibition activity of the enterocins, 20 mL of each CFS from the *Enterococcus* strains was added to BHI broth (150 mL) (Sigma‐Aldrich) containing *L. monocytogenes* ATCC 7644 in the early exponential phase (3 h). The medium was incubated at 37°C for 15 h, and the absorbance values (OD_600_) of the medium were determined every hour using a plate reader (Epoch, BioTek, Agilent Technologies Inc., Santa Clara, California, USA). Medium without the CFS was used as a control (Pinto et al. 2009).

Nine different treatments were used for in situ inhibition of *L. monocytogenes* , including *L. monocytogenes* alone (positive control) (trial 1), *L. monocytogenes* + Enterocin BH04 (trial 2), *L. monocytogenes* + Enterocin BH12 (trial 3), *L. monocytogenes* + Enterocin BH84 (trial 4), *L. monocytogenes* + Enterocin BH99 (trial 5), *L. monocytogenes* + *E. faecium* BH04 (trial 6), *L. monocytogenes* + *E. faecium* BH12 (trial 7), *L. monocytogenes* + *E. faecium* BH84 (trial 8), and *L. monocytogenes* + *E. faecium* BH99 (trial 9). Fresh cultures of *L. monocytogenes* and *E. faecium* were inoculated into reconstituted skim milk (RSM) at approximately 10^5^ cfu/mL, while the bacteriocins were inoculated at 10^3^ AU/mL. The RSM was then incubated at 37°C for 15 h and viable cell counts were determined every 3 h. For the enumeration of *L. monocytogenes* , PALCAM agar (Merck) with *Listeria* selective supplement (Merck) was used, and the plates were incubated at 37°C for 24 h (Benkerroum et al. 2002).

### 2.6 Determining Genes Encoding Enterocin Production and Sequencing

Genes coding for enterocin production were detected by polymerase chain reaction (PCR) using primers specific for *entA*, *entB*, *entP*, and *entL50* as presented in table 2 (Du Toit et al. 2000). The genomic DNA of the *Enterococcus* strains was obtained using the method of Singh and Ramesh (2009). The PCR was performed with 25 μL Taq OptiMix CLEAR Master Mix (AMPLIQON, Odense, Denmark), 1 μL of each primer (forward and reverse), 50 ng/μL template DNA, and nuclease‐free water (Thermo Fisher Scientific, Waltham, Massachusetts, USA). The thermal cycling was as follows: pre‐denaturation at 95°C for 5 min, denaturation at 95°C for 30 s, annealing at the correct temperature (see Table 2) for 40 s, and extension at 72°C for 30 s, for a total of 30 cycles, and post‐extension at 72°C for 5 min. The PCR products were then visualized using ClearBand Safe DNA Gel Stain Solution (Ecotech Biotechnology, Erzurum, Turkey) in 1.5% (w/v) agarose gel under UV light. Genes were sequenced using the ABI 3130 Xl genetic analyzer (Applied Biosystems, Waltham, Massachusetts, USA).

**TABLE 2:** The primers used for determining genes encoding enterocin production.

The primer name	Sequence (5′‐3′)	Target gene	Annealing temperature (°C)	Amplicon size (bp)	Reference
Enterocin A	F: AAATATTATGGAAATGGAGTGTAT	*entA*	55.5	125	Du Toit et al. (2000)
	R: GCACTTCCCTGGAATTGCTC				
Enterocin B	F: GAAAATGATCACAGAATGCCTA	*entB*	54.5	159	
	R: GTTGCATTTAGAGTATACATTTG				
Enterocin P	F: TATGGTAATGGTGTTTATTGTAAT	*entP*	54.5	112	
	R: ATGTCCCATACCTGCCAAAC				
Enterocin L50	F: STGGGAGCAATCGCAAAATTAG	*entL50*	56.5	135	
	R: ATTGCCCATCCTTCTCCAAT				

### 2.7 Characterization of Enterocins

The CFS containing enterocins produced by the *Enterococcus* spp. was obtained from M17 broth (Merck) containing 0.5% glucose (Merck) for characterization of the enterocins. The CFS was exposed to the following treatments. At the end of all treatments, the percent residual activity (%) of the enterocins was calculated by the two‐fold serial dilution method on BHIA (Sigma‐Aldrich) seeded with *L. monocytogenes* ATCC 7644.

#### 2.7.1 Temperature Resistance

The CFS was exposed to different temperatures including −80°C, −20°C, 4°C, 37°C, 60°C, 80°C, and 100°C for 10, 30, and 60 min and 121°C for 15 min to determine the cooling and heating resistance of the enterocins (Ogunbanwo et al. 2003).

#### 2.7.2 pH Sensitivity

The pH of the CFS was adjusted to 2, 4, 6, 8, 10, and 12 using 1 N NaOH (Merck) or HCl (Merck) and incubated at room temperature (25°C) for 3 h. The pH of the CFS was then neutralized to eliminate the antimicrobial activity of acidic or basic CFS (Meral‐Aktaş 2023).

#### 2.7.3 Enzyme Treatment

The enterocins‐containing CFS was tested for sensitivity to various enzymes. For this purpose, enzymes including catalase, lysozyme, pepsin, proteinase K, and trypsin (Sigma‐Aldrich) were separately added to the CFS at a concentration of 1 mg/mL. The CFS was then incubated at 37°C for 2 h and heated at 80°C for 10 min to inactivate the enzymes (Todorov and Dicks 2005).

#### 2.7.4 Effect of Chemicals on Enterocin Activity

To determine the effects of some chemicals on enterocin activity, 2‐mercaptoethanol, acetone, chloroform, ethanol, ethylenediaminetetraacetic acid (EDTA), glycerol, methanol, sodium chloride (NaCl), sodium dodecyl sulfate (SDS), triton X‐100, Tween‐20, Tween‐80, urea (Sigma‐Aldrich) and reconstituted skim milk were added to the CFS at a final concentration of 1% (v/v). The CFS was then incubated at 37°C for 5 h (Todorov and Dicks 2005).

#### 2.7.5 Effect of UV Light on Enterocin Activity

The enterocins were exposed to UV light to determine their sensitivity. The CFS was held under UV light (distance: 30 cm) for 5 min (Ogunbanwo et al. 2003).

### 2.8 Statistical Analysis

The experiments were performed in triplicate, and all results are expressed as mean ± standard deviation (Kenward 1987). All means and standard deviations were calculated using the SPSS version 20.0 package program (SPSS Inc., Chicago, IL, USA).

## 3 Results and Discussion

### 3.1 Microbial Growth Curve and Bacteriocin Production

**FIGURE 1:**
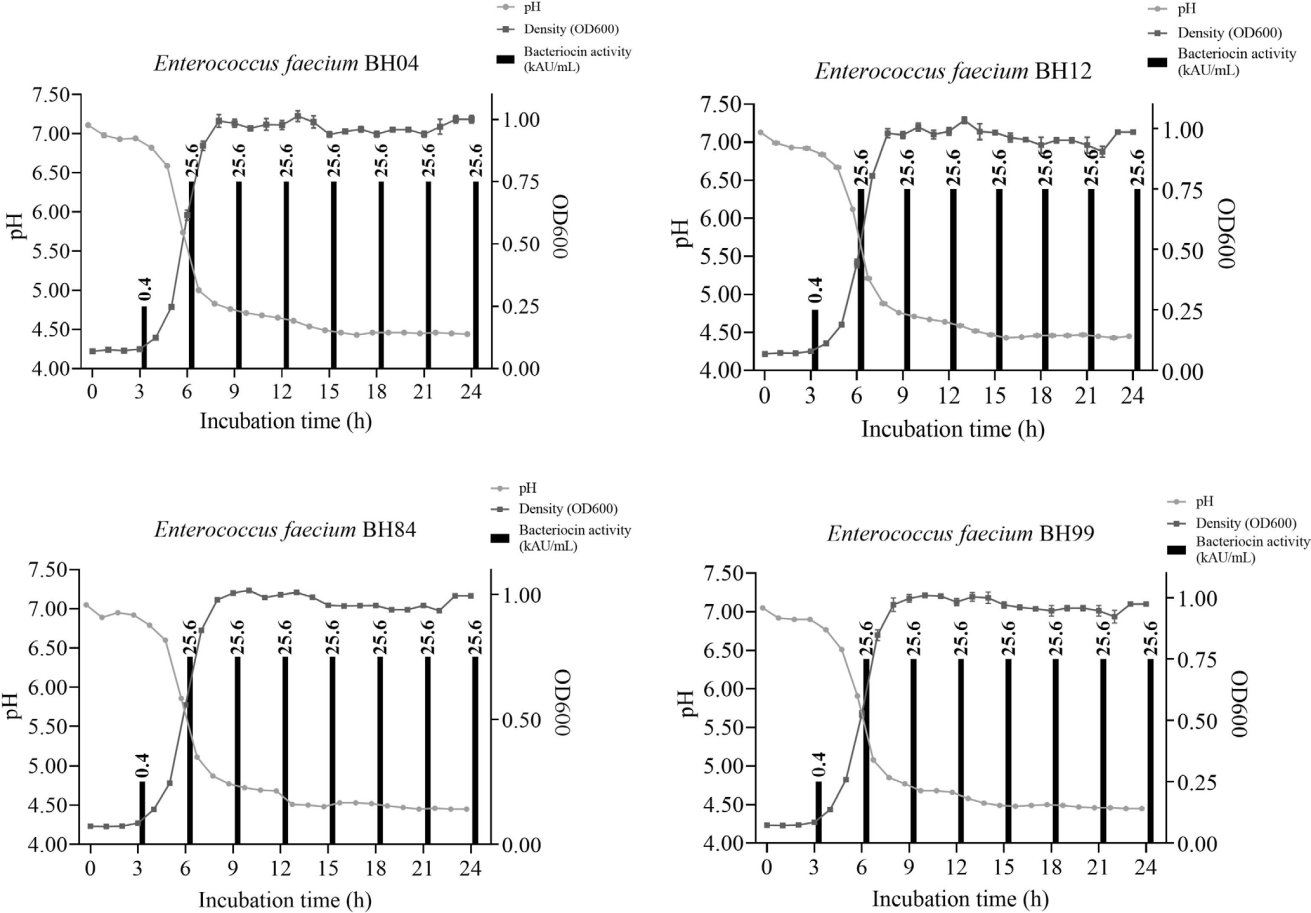
Microbial growth curves and enterocin production abilities of the *Enterococcus faecium* strains. 1 kAU/mL indicates 1000 AU/mL.

During microbial growth, the pH of the medium or food decreases and the microbial biomass increases. In addition, it is known that some microorganisms can produce antimicrobial peptides such as bacteriocin with their growth (Yang et al. 2018). In this study, microbial growth, including pH and optical density, and enterocin production by the *Enterococcus* spp. are shown in Figure 1. Considering the pH results, all strains decreased the pH of M17 broth (Merck) from 7.13 to 4.53 within 24 h. Vandera et al. (2020) reported that *Enterococcus* spp. isolates decreased the pH of M17 broth to 5.51 at the end of the incubation at 30°C for 24 h. On the other hand, Meral‐Aktaş, Erdoğan, and Çeti̇n (2024) found that *E. faecium* strains reduced the pH of M17 broth containing glucose to 4.50–5.00 (at 37°C for 24 h). Differences in results may be due to pre‐incubation treatment, differences in incubation temperature, and the addition of glucose. In this context, the OD_600_ value increased from 0.068 to 1.034 and the OD_600_ curve clearly showed the microbial growth phases of the strains, including the lag, exponential, and stationary phases. The results concluded that all strains started to produce enterocin with an activity of 0.4 kAU/mL (400 AU/mL) after 3 h. Furthermore, the bacteriocin activity increased to 25.6 kAU/mL (25,600 AU/mL) after 6 h and was maintained throughout the incubation period (Figure S1). Yang et al. (2018) found that the CFS of *E. faecium* JFR‐1 grown in the De Man, Rogosa, Sharpe (MRS) broth had bacteriocin activity against *L. monocytogenes* with an activity of 900 AU/mL after 11 h. In addition, Nascimento et al. (2010) found that the bacteriocin activity of *E. faecium* FAIR‐E 198 grown in MRS broth varied from 1600 to 19,200 AU/mL against different *L. monocytogenes* strains. These results suggest that the enterocin activity may be affected by differences in the medium, *E. faecium* strains, and *L. monocytogenes* strains. On the other hand, there are many studies reporting that low pH may reduce the activity of bacteriocins by microorganisms (Nascimento et al. 2010; Ben Belgacem et al. 2012; Yang et al. 2018). However, in this study, the activity of the enterocins produced by the *E. faecium* strains was not affected by decreasing pH during the incubation period. Furthermore, the strains rapidly produced enterocins with inhibitory activity against *L. monocytogenes* ATCC 7644 during the incubation. This study showed that the strains and/or their enterocins may have potential as antilisterial agents due to their rapid production and stability at low pH.

### 3.2 Antibacterial Spectrum of Bacteriocins

Bacteriocins produced by LAB generally have many applications in food biotechnology due to their heat stability, inhibition effect on saprophytes and/or pathogens in products, and suppression of uncontrolled growth of starter cultures (Cotter et al. 2005). In addition, bacteriocins are known to have a broad inhibitory effect on Gram‐positive bacteria (De Kwaadsteniet et al. 2005). Although not as broad as the effect on Gram‐positive bacteria, some bacteriocins have also been reported to have an effect on Gram‐negative bacteria. For example, De Kwaadsteniet et al. (2005) reported that bacteriocins from *Enterococcus mundtii* ST15 inhibited Gram‐negative bacteria such as *Acinetobacter baumannii*, *Klebsiella pneumoniae* , and *Pseudomonas aeruginosa* , while Woraprayote et al. (2015) reported that bacteriocins from *Weissella hellenica* BCC 7293 inhibited *Aeromonas hydrophila* , *Escherichia coli* , *Pseudomonas aeruginosa* , and *Salmonella Typhimurium* . In addition, Wang et al. (2022) showed that the combination of bacteriocins by LAB with chelators such as lactic acid could increase the inhibitory effect on Gram‐negative bacteria. Therefore, the antibacterial activity of the bacteriocins should be investigated in a broad spectrum. In this study, the antibacterial spectrum of the enterocins obtained from *E. faecium* strains against some pathogens and LAB was determined, and the results are given in Table 1. All the strains had an inhibitory effect on *L. monocytogenes* ATCC 7644 ranging from 19.3 to 20.5 mm (Figure S2) as reported by García et al. (2004), Meral‐Aktaş (2023) and Azevedo et al. (2024). In addition, the strains also inhibited the growth of *Heyndrickxia coagulans* ATCC 7050 (previously named *Bacillus coagulans*, Narsing Rao et al. (2023)) between 10.0 and 12.5 mm. Similar results were reported by García et al. (2003) and Lucas et al. (2006). The results showed that *E. faecium* BH84 and BH99 had an inhibitory effect on *Lactobacillus delbrueckii* subsp. *bulgaricus* 239 M (21.0 mm), while *E. faecium* BH08 and BH12 did not. This was an expected result, as *Lactobacillus delbrueckii* subsp. *bulgaricus* is known to be susceptible to bacteriocins (Hegarty et al. 2017). On the other hand, other *Lactobacillus* spp. used for antibacterial spectrum, including *Lactobacillus helveticus* ATCC 15009 and *Lactobacillus casei* ATCC 393, were not affected by enterocins. Therefore, it can be concluded that the enterocins may be important for both food safety and food quality due to their inhibitory effect on *L. monocytogenes* ATCC 7644.

### 3.3 In Vitro (Mode of Action) and In Situ (Milk Model) Inhibition of *Listeria monocytogenes*

**FIGURE 2:**
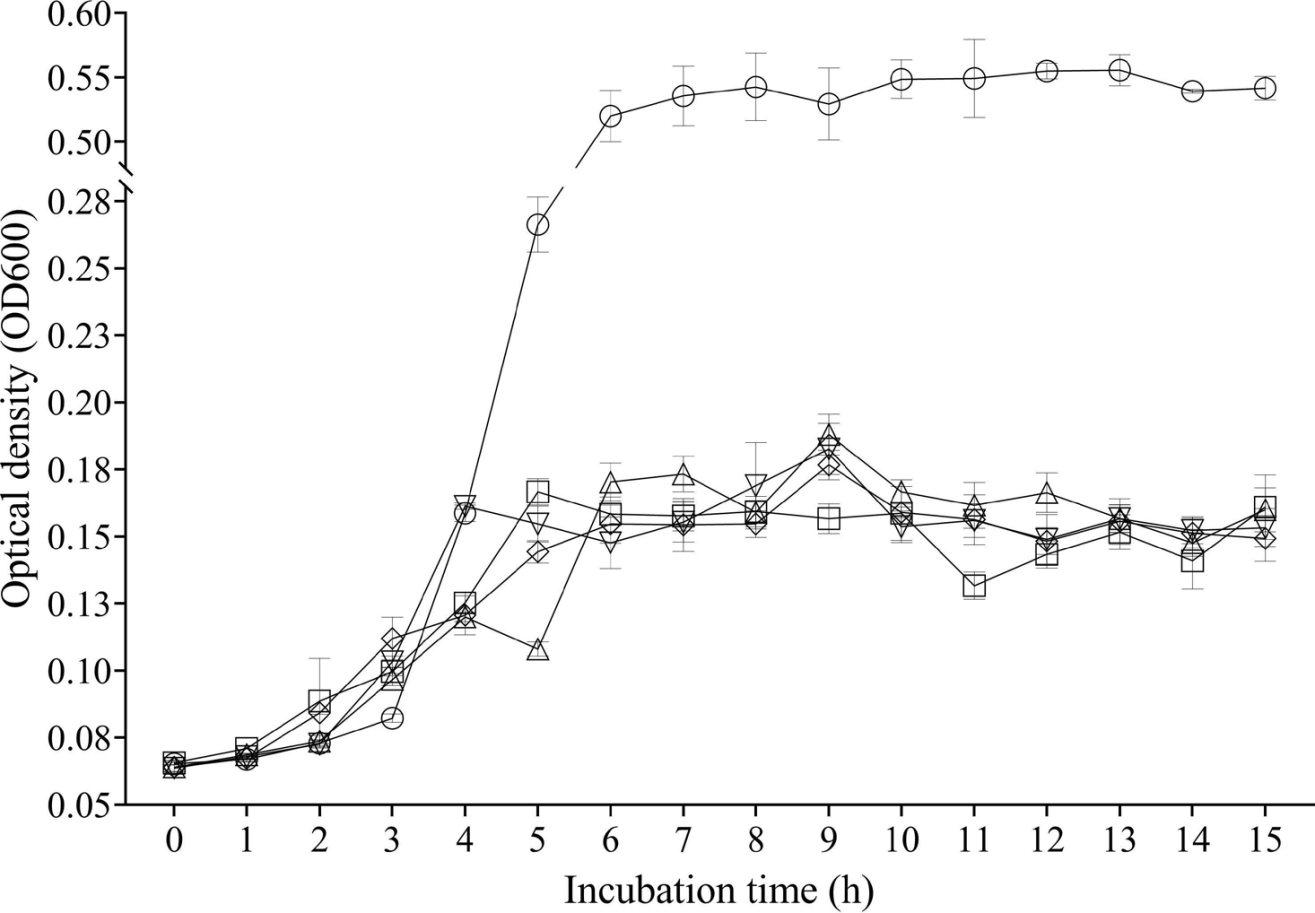
The growth of *L. monocytogenes* ATCC 7644 in BHI broth containing enterocins. ○: *L. monocytogenes* ATCC 7644 alone, □: *L. monocytogenes* ATCC 7644 + enterocin BH04, ∆: *L. monocytogenes* ATCC 7644 + enterocin BH12, ▽: *L. monocytogenes* ATCC 7644 + enterocin BH84, ◊: *L. monocytogenes* ATCC 7644 + enterocin BH99.

In this study, the in vitro inhibition effect of enterocin BH04, BH12, BH84, and BH99 on *L. monocytogenes* ATCC 7644 was investigated, and the results obtained are shown in Figure 2 and Table S2. The OD_600_ value of BHI with *L. monocytogenes* ATCC 7644 alone varied from 0.07 to 0.54 over the incubation time. On the other hand, the OD_600_ value of BHI with *L. monocytogenes* ATCC 7644 and enterocin BH04, BH12, BH84, and BH99 increased up to 0.16 at the end of 15 h. It was concluded that all enterocins used in this study were able to inhibit the growth of *L. monocytogenes* ATCC 7644 in BHI compared to the control culture sample containing the pathogen growing alone. Similarly, there are many studies reporting that the enterocins obtained from *E. faecium* strains can exhibit a bacteriostatic mode of action on *L. monocytogenes* (Chakchouk‐Mtibaa et al. 2014; Yang and Moon 2021; Du et al. 2022).

**FIGURE 3:**
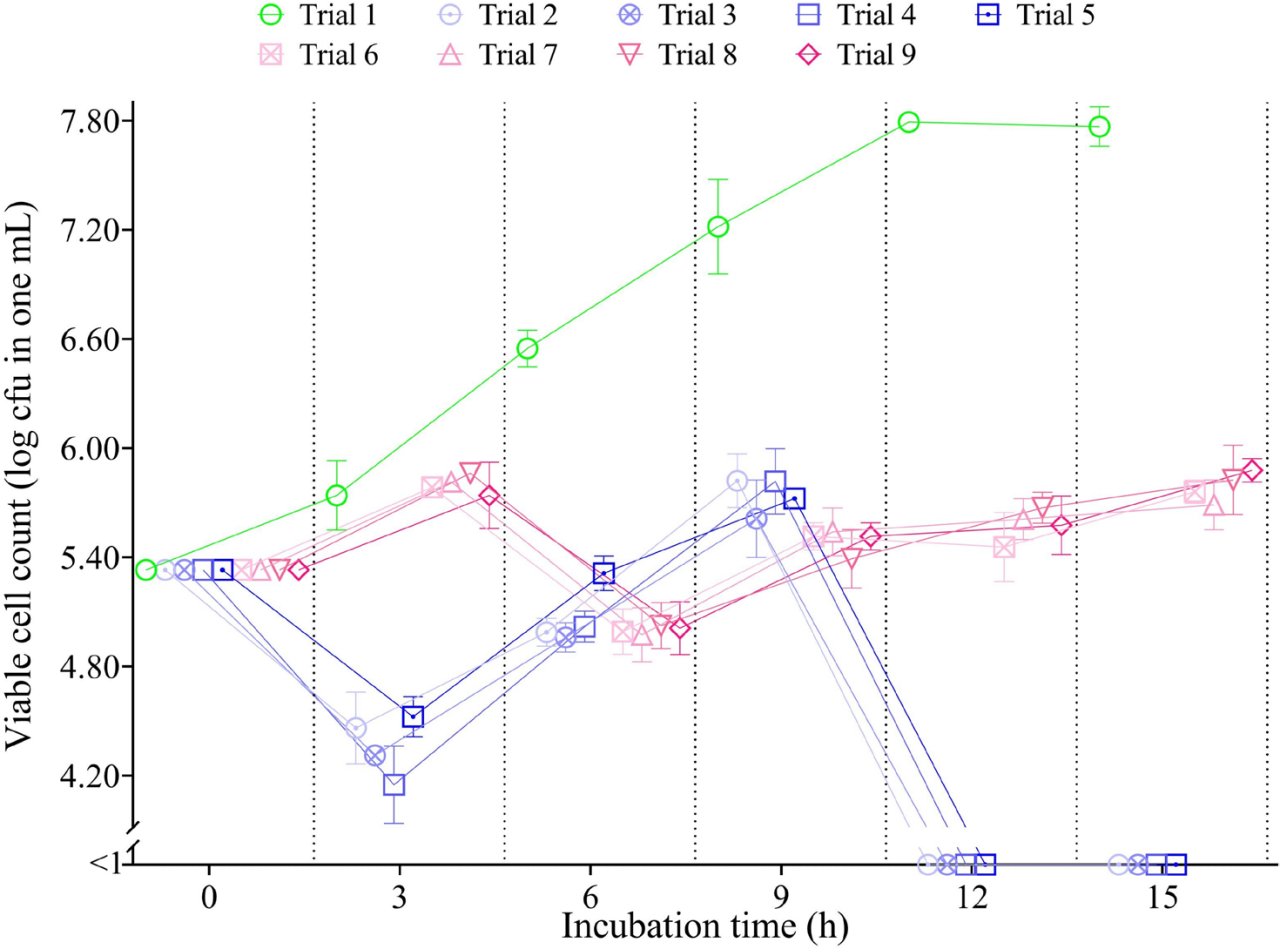
In situ inhibition of *L. monocytogenes* ATCC 7644 in RSM. Cfu: Colony‐forming unit, h: Hour. Trial 1: *L. monocytogenes* alone (positive control), trial 2: *L. monocytogenes* + Enterocin BH04, trial 3: *L. monocytogenes* + Enterocin BH12, trial 4: *L. monocytogenes* + Enterocin BH84, trial 5: *L. monocytogenes* + Enterocin BH99, trial 6: *L. monocytogenes* + *E. faecium* BH04, trial 7: *L. monocytogenes* + *E. faecium* BH12, trial 8: *L. monocytogenes* + *E. faecium* BH84, trial 9: *L. monocytogenes* + *E. faecium* BH99.

The results of the in situ inhibitory activity of enterocin BH04, BH12, BH84, and BH99 on *L. monocytogenes* ATCC 7644 in the RSM are presented in Figure 3 and Table S3. The count of *L. monocytogenes* ATCC 7644 alone increased from 5.33 to 7.77 log cfu/mL at the end of 15 h. However, the viable cell count of the pathogen in the RSM combined with *E. faecium* BH04, BH12, BH84, and BH99 was between 5.59 and 5.96 log cfu/mL at the end of the incubation time. The *E. faecium* strains were bacteriostatic against *L. monocytogenes* ATCC 7644 in the RSM model. In addition, the count of *L. monocytogenes* ATCC 7644 in RSM containing enterocins BH04, BH12, BH84, and BH99 fell below the countable limit by the twelfth hour of incubation. It was concluded that all enterocins inhibited *L. monocytogenes* ATCC 7644 and had bactericidal activity against the pathogen. Similarly, Nascimento et al. (2010) reported that *E. faecium* FAIR‐E 198 strain had bactericidal activity against *L. monocytogenes* Scott A in the skimmed milk. Furthermore, in the study by Vandera et al. (2017), in skimmed milk (pH decreased from 6.42 to 6.04 during incubation) containing enterocin‐producing *E. faecium* KE82, it was found that the count of *L. monocytogenes* was suppressed (4.23 log cfu/mL) after 6 h of incubation compared to the control (5.21 log cfu/mL), and this difference was statistically significant.

The results of the in vitro and in situ inhibition experiments indicated that enterocin BH04, BH12, BH84, and BH99 could be used as antilisterial agents. Moreover, *E. faecium* BH04, BH12, BH84, and BH99 strains were also able to suppress the growth of *L. monocytogenes* ATCC 7644. Therefore, it is thought that these strains can be used as a co‐culture to control the pathogens.

### 3.4 Determining Genes Encoding Enterocin Production and Sequencing

**FIGURE 4:**
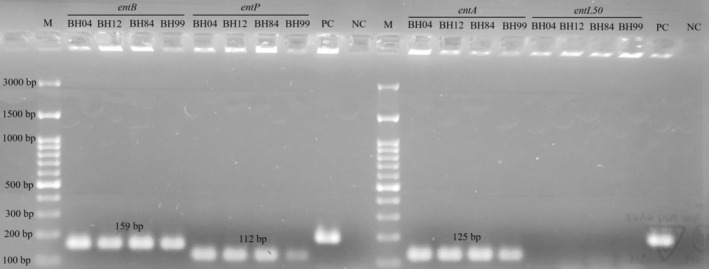
The gel image of genes encoding enterocin production, including *entA*, *entB*, *entP*, and *entL50*, in *Enterococcus faecium* BH04, BH12, BH84, and BH99 strains. M: DNA ladder, bp: Base pair, PC: Positive control, NC: Negative control.

**FIGURE 5:**
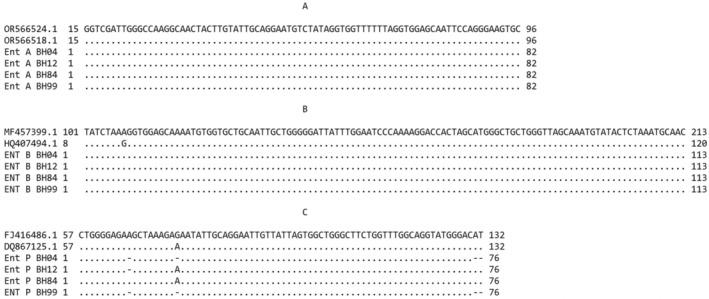
Nucleotide sequencing of *entA* (A), *entB* (B), *entP* (C) genes of the strains.

In this study, *entA*, *entB*, *entP*, and *entL50* genes encoding enterocin production were screened by PCR. The resulting gel images are shown in Figure 4. The results showed that all the strains used in this study had *entA*, *entB*, and *entP* genes as in the study by Meral‐Aktaş, Erdoğan, and Çeti̇n (2024). On the contrary, the *entL50* gene was not detected in any of the strains. In addition, the nucleotide sequencing of *entA*, *entB*, and *entP* genes of the strains is shown in Figure 5. *entA* and *entB* genes in all strains had 100% identity with each other and with *entA* (OR566524.1 and OR566518.1) and *entB* (MF457399.1) genes in the National Center for Biotechnology Information (NCBI). On the other hand, *entP* genes in all strains had up to four nucleotide gaps. *entP* genes were 98.68% identity with FJ416486.1 and 100% identity with DQ867125.1 when these gaps were ignored.

### 3.5 Characterization of Enterocins

In this study, enterocin BH04, BH12, BH84, and BH99 were characterized with respect to different treatments including temperature, pH, enzyme, chemical, and UV. The characterization results are shown in Table 3. Cold storage (−80°C, −20°C, and +4°C) and hot storage (37°C, 60°C, and 80°C) did not affect the antilisterial activity of enterocins. On the other hand, the residual activity decreased by 50% after 30 and 60 min at 100°C, whereas it did not change after 10 min at 100°C. In addition, the inhibitory effect of all enterocins on *L. monocytogenes* ATCC 7644 was significantly reduced after sterilization (at 121°C for 15 min). Bacteriocins are known to have a protein structure and to be heat‐stable (Gálvez et al. 2007). The present study revealed that the enterocins were stable against cold storage and heating up to 100°C for 10 min. Similar results were also reported by Mareková et al. (2007) and Meral‐Aktaş (2023).

**TABLE 3:** Characterization of enterocin BH04, BH12, BH84, and BH99 to different treatments.

Treatment	Time (min)	Residual activity (%)			
		Enterocin BH04	Enterocin BH12	Enterocin BH84	Enterocin BH99
Temperature					
−80°C	10	100	100	100	100
	30	100	100	100	100
	60	100	100	100	100
−20°C	10	100	100	100	100
	30	100	100	100	100
	60	100	100	100	100
4°C	10	100	100	100	100
	30	100	100	100	100
	60	100	100	100	100
37°C	10	100	100	100	100
	30	100	100	100	100
	60	100	100	100	100
60°C	10	100	100	100	100
	30	100	100	100	100
	60	100	100	100	100
80°C	10	100	100	100	100
	30	100	100	100	100
	60	100	100	100	100
100°C	10	100	100	100	100
	30	50	50	50	50
	60	50	50	50	50
121°C	15	6.25	3.125	6.25	6.25
pH					
2		100	100	100	100
4		100	100	100	100
6		100	100	100	100
8		100	100	100	100
10		100	100	100	100
12		12.5	12.5	6.25	6.25
Enzyme (1 mg/mL)					
Catalase		100	100	100	100
Lysozyme		100	100	100	100
Pepsin		100	25	12.5	6.25
Proteinase K		0	0	0	0
Trypsin		0	0	0	0
Chemicals (1%, v/v)					
2‐mercaptoethanol		100	100	100	100
Acetone		100	100	100	100
Chloroform		100	100	100	100
Ethanol		400	400	200	200
Ethylenediaminetetraacetic acid (EDTA)		100	100	100	100
Glycerol		100	100	100	100
Methanol		200	200	200	400
Reconstitute skim milk		100	100	100	100
Sodium chloride (NaCl)		100	100	100	100
Sodium dodecyl sulfate (SDS)		200	400	400	200
Triton X‐100		100	100	100	100
Tween‐20		100	100	100	100
Tween‐80		100	100	100	100
Urea		100	100	100	100
UV (30 cm, 5 min)					
UV		50	25	50	50

The antilisterial activity of enterocin BH04, BH12, BH84, and BH99 was not affected by pH 2, 4, 6, 8, and 10 treatments as reported by Qiao et al. (2020). However, in the study by Du et al. (2022), the residual activity of enterocin HDX‐2 obtained from *E. faecium* HDX‐2 at pH 10 was found to be reduced to 80.28%. Considering these results, it can be reported that the stability of enterocins BH04, BH12, BH84, and BH99 is higher in alkaline medium. However, the enterocins used in this study lost about 90% of their antilisterial activity at pH 12. Similarly, Yang and Moon (2021) reported that the activity of enterocin from *E. faecium* CJNU 2524 decreased by 50% in pH 11. All these results revealed that the enterocins may lose their antilisterial activity in environments above pH 10.

The inhibitory effect of the enterocins used in this study on *L. monocytogenes* ATCC 7644 was not altered by the addition of catalase and lysozyme. Similarly, Franz et al. (1996) found that enterocin 900 from *E. faecium* BFE 900 was stable to catalase and lysozyme. On the other hand, enterocins are known to be sensitive to some proteolytic enzymes such as pepsin, proteinase K, and trypsin (Franz et al. 1996; Kumar et al. 2010; Qiao et al. 2020). The results of this study showed that the enterocins were sensitive to pepsin, proteinase K, and trypsin (except the enterocin BH04 against pepsin).

All enterocins were stable to all chemicals used in this study. In addition, ethanol, methanol, and SDS increased the antilisterial activity of the enterocins by between 200% and 400%. These results were expected because ethanol (Oh and Marshall 1993) and methanol (Sidorenko and Buzoleva 2012) have antimicrobial activity against *L. monocytogenes* . In addition, SDS is known to have a potentiating or antimicrobial effect (Kaktcham et al. 2019). Therefore, similar results were found in the present study.

In this study, UV treatment reduced antilisterial activity of enterocins by 50% for BH04, BH84, and BH99 and by 75% for BH12. Ogunbanwo et al. (2003) reported that bacteriocins produced by *Lactobacillus plantarum* F1 and *Lactobacillus brevis* OG1 were not affected by UV light. It is suggested that these different results may be due to the type of bacteriocin.

## 4 Conclusion

This study demonstrated that *E. faecium* BH04, BH12, BH84, and BH99 have the potential to produce enterocins effective against *L. monocytogenes* ATCC 7644, an important foodborne pathogen. The bactericidal and bacteriostatic activity of enterocin BH04, BH12, BH84, and BH99 against *L. monocytogenes* ATCC 7644 by strains carrying *entA*, *entB*, and *entP* genes encoding enterocin production was demonstrated using in vitro mode of action and in situ milk model. In addition, the enterocins retained their antilisterial activity under certain conditions such as heating and acidic environment. Finally, because of all this and because the enterocins were effective against *L. monocytogenes* ATCC 7644 but had no inhibitory effect on LAB such as *Streptococcus thermophilus* 212S, *Lactobacillus helveticus* ATCC 15009, and *Lactobacillus casei* ATCC 393, it was concluded that these strains or their bacteriocins could be used as antilisterial agents in fermented products.

## Conflicts of Interest

The author declares no conflicts of interest.

## Supporting information


Data S1.

## Data Availability

Data available on request due to privacy/ethical restrictions.
