# Supplementary material for: Characterisation of Enterocins Produced by Antilisterial Enterococcus faecium BH04, BH12, BH84, and BH99 and In Vitro/In Situ Inhibition of Listeria monocytogenes
Source: Food Sci Nutr. 2025 Apr 1;13(4):e70142. doi: 10.1002/fsn3.70142 (PMC11961377; doi:10.1002/fsn3.70142)
Supplement: Supplementary file 1 — Data S1. [file FSN3-13-e70142-s001.docx]

**Supplementary Material:** Characterisation of Enterocins Produced by Antilisterial *Enterococcus* *faecium* BH04, BH12, BH84, and BH99 and *In vitro*/*In situ* Inhibition of *Listeria monocytogenes*


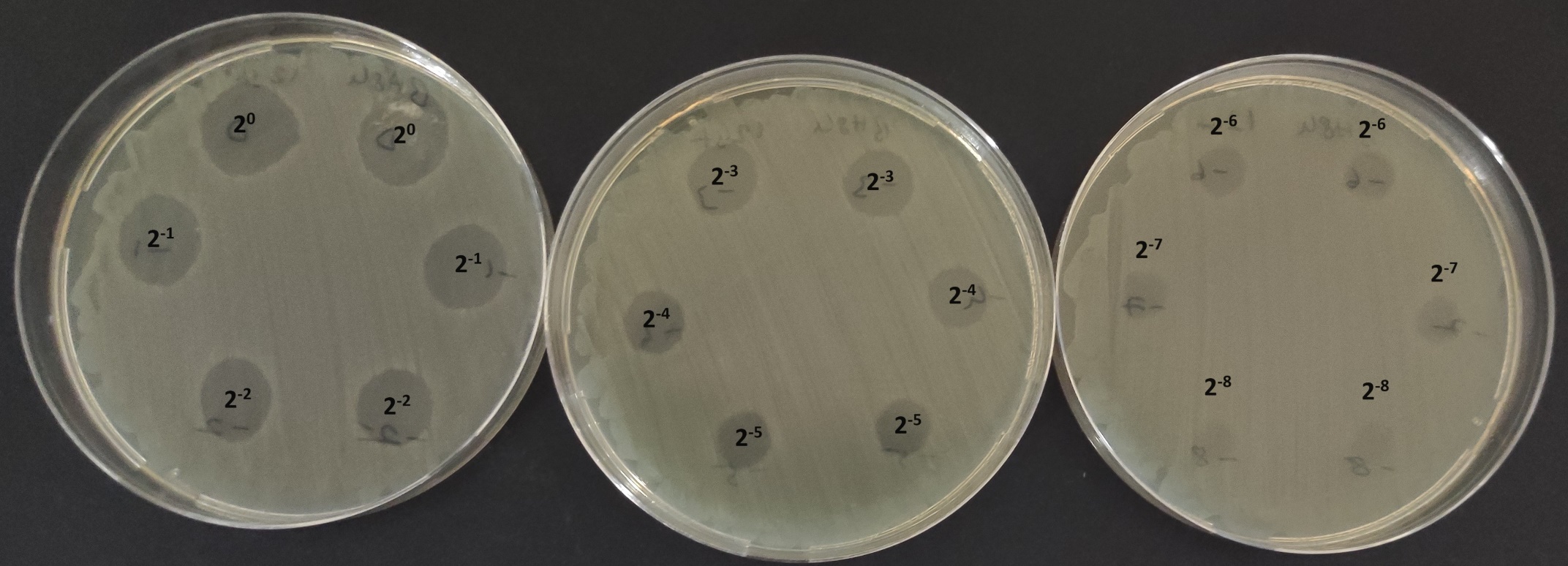


**Figure S1.** Inhibitory effect of the enterocin BH84 on *Listeria monocytogenes* ATCC 7644 by the two-fold serial dilution method


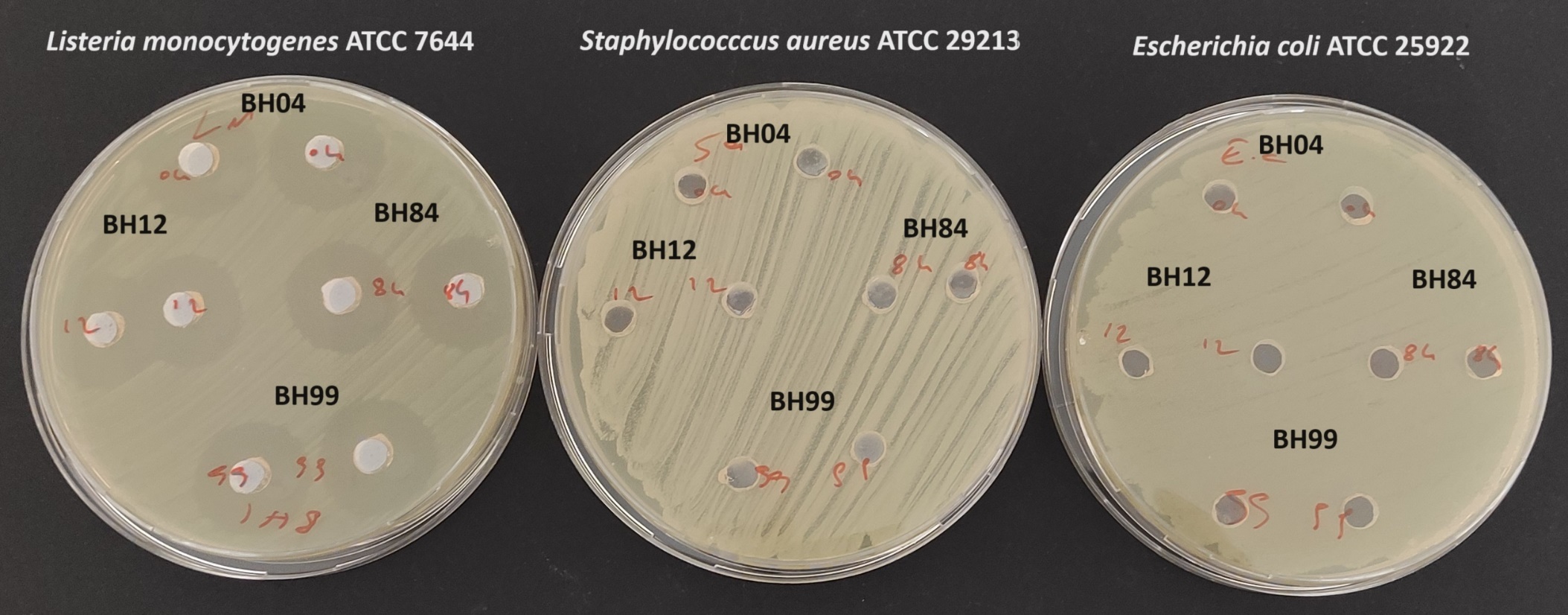


**Figure S2.** Antibacterial spectrum of the enterocins produced by the *Enterococcus* strains on *Listeria monocytogenes* ATCC 7644, *Staphylococccus aureus* ATCC 29213, and *Escherichia coli* ATCC 25922

**Table S1.** 16S rRNA identification, probiotic characterisation and safety assessment results of *Enterococcus faecium* BH04, BH12, BH84 and BH99 used in this study (Çetin and Aktaş, 2024)^a^

| Results | | Strains | | | |
| --- | --- | --- | --- | --- | --- |
|  |  | *Enterococcus faecium* BH04 | *Enterococcus faecium* BH12 | *Enterococcus faecium* BH84 | *Enterococcus faecium* BH99 |
| 16S rRNA identification results | Sequence ID (Similarity, %) | OR551443.1 (100%) | OR551443.1 (100%) | OR551443.1 (100%) | OR551443.1 (100%) |
| Probiotic characterisation | Tolerance to gastric juice (%) | 79.25±0.49 | 82.05±0.78 | 76.80±1.56 | 82.05±0.35 |
|  | Tolerance to 1% bile salts (%) | 81.76±7.59 | 77.21±0.04 | 103.84±11.98 | 69.36±0.93 |
|  | Antimicrobial activity against *L. monocytogenes* ATCC 7644 | + | + | + | + |
|  | Bile salt hydrolase property | Strong | Strong | Strong | Strong |
|  | *In vitro* cholesterol lowering potential (%) | 70.45±0.99 | 69.47±0.79 | 95.00±0.24 | 95.00±0.24 |
| Safety assessment | Antibiotics to which the strains are resistant | Gentamicin, 10 μg  Kanamycin, 30 μg  Streptomycin, 25 μg | Gentamicin, 10 μg  Kanamycin, 30 μg  Streptomycin, 25 μg | Gentamicin, 10 μg  Kanamycin, 30 μg  Streptomycin, 25 μg | Gentamicin, 10 μg  Kanamycin, 30 μg  Streptomycin, 25 μg |
|  | Virulence genes possessed by strains | *gelE* | *gelE* | *esp* | *gelE* |
|  | Haemolytic activity | - | - | - | - |
|  | DNase activity | - | - | - | - |
|  | Galleria mellonella mortality (%) | 0 | 0 | 0 | 0 |
|  | Gelatinase activity | - | - | - | - |
|  | Biofilm formation | Non or weak | Non or weak | Non or weak | Non or weak |

^a^ All results are presented as mean±standard deviation. The strains were tested for resistance to the antibiotics ampicillin, vancomycin, gentamicin, kanamycin, streptomycin, erythromycin, clindamycin, tetracycline, and chloramphenicol. The strains were analysed for virulence genes such as *ace* (adhesion to collagen), *asa* (aggregation substance), *cylA* (cytolisin), *efaAfs* (*Enterococcus faecalis* specific endocarditis antigen), *esp* (enterococcal surface protein), *gelE* (gelatinase), and *hyl* (hyaluronidase).

**Table S2.** The growth of *Listeria monocytogenes* ATCC 7644 in brain heart infusion broth containing enterocin BH04, BH12, BH84, and BH99^a^

| Incubation time (hour) | The growth of *L. monocytogenes* ATCC 7644 in BHI broth containing enterocin BH04, BH12, BH84, and BH99 (Optical density, OD_600_) | | | | | Sig. |
| --- | --- | --- | --- | --- | --- | --- |
|  | ATCC 7644 | ATCC 7644 + enterocin BH04 | ATCC 7644 + enterocin BH12 | ATCC 7644 + enterocin BH84 | ATCC 7644 + enterocin BH99 |  |
| 1 | 0.067±0.001^b^ | 0.071±0.000^a^ | 0.069±0.001^b^ | 0.068±0.001^b^ | 0.067±0.001^b^ | ** |
| 2 | 0.073±0.001^a^ | 0.089±0.016^a^ | 0.074±0.002^a^ | 0.073±0.002^a^ | 0.084±0.001^a^ | ns |
| 3 | 0.082±0.002^c^ | 0.100±0.002^b^ | 0.097±0.002^b^ | 0.103±0.002^b^ | 0.112±0.008^a^ | *** |
| 4 | 0.159±0.003^a^ | 0.125±0.004^b^ | 0.120±0.002^b^ | 0.161±0.001^a^ | 0.121±0.007^b^ | *** |
| 5 | 0.266±0.011^a^ | 0.167±0.005^b^ | 0.108±0.003^d^ | 0.155±0.007^c^ | 0.144±0.004^c^ | *** |
| 6 | 0.520±0.020^a^ | 0.158±0.006^bc^ | 0.170±0.007^b^ | 0.148±0.010^c^ | 0.155±0.007^bc^ | *** |
| 7 | 0.536±0.023^a^ | 0.158±0.006^b^ | 0.173±0.007^b^ | 0.155±0.007^b^ | 0.154±0.010^b^ | *** |
| 8 | 0.543±0.026^a^ | 0.159±0.006^b^ | 0.159±0.004^b^ | 0.169±0.016^b^ | 0.155±0.005^b^ | *** |
| 9 | 0.529±0.028^a^ | 0.157±0.006^c^ | 0.188±0.008^b^ | 0.183±0.010^bc^ | 0.177±0.006^bc^ | *** |
| 10 | 0.549±0.015^a^ | 0.159±0.010^b^ | 0.167±0.005^b^ | 0.154±0.006^b^ | 0.159±0.002^b^ | *** |
| 11 | 0.549±0.030^a^ | 0.132±0.005^c^ | 0.162±0.009^b^ | 0.156±0.006^bc^ | 0.156±0.009^bc^ | *** |
| 12 | 0.555±0.006^a^ | 0.143±0.005^c^ | 0.166±0.007^b^ | 0.149±0.009^c^ | 0.148±0.005^c^ | *** |
| 13 | 0.556±0.012^a^ | 0.152±0.006^b^ | 0.157±0.005^b^ | 0.157±0.005^b^ | 0.156±0.009^b^ | *** |
| 14 | 0.539±0.002^a^ | 0.141±0.010^b^ | 0.148±0.005^b^ | 0.152±0.005^b^ | 0.151±0.006^b^ | *** |
| 15 | 0.542±0.009^a^ | 0.161±0.012^b^ | 0.160±0.008^b^ | 0.153±0.007^b^ | 0.149±0.009^b^ | *** |

^a^ ATCC 7644 indicates *Listeria monocytogenes* ATCC 7644. Sig.: Degree of statistical significance, ns: Not statistically significant, *: p<0.05, **: p<0.01, ***: p<0.001. Lowercase letters represent statistical differences in the same row.

**Table S3.** *In situ* inhibition of *Listeria monocytogenes* ATCC 7644 in the reconstituted skim milk^a^

| Incubation time (hour) | Viable cell count of *L. monocytogenes* ATCC 7644 in the reconstituted skim milk (log colony-forming unit/mL) | | | | | | | | | Sig. |
| --- | --- | --- | --- | --- | --- | --- | --- | --- | --- | --- |
|  | Trial 1 | Trial 2 | Trial 3 | Trial 4 | Trial 5 | Trial 6 | Trial 7 | Trial 8 | Trial 9 |  |
| 0 | 5.33±0.04^a^ | 5.33±0.04^a^ | 5.33±0.04^a^ | 5.33±0.04^a^ | 5.33±0.04^a^ | 5.33±0.04^a^ | 5.33±0.04^a^ | 5.33±0.04^a^ | 5.33±0.04^a^ | ns |
| 3 | 5.74±0.19^a^ | 4.46±0.20^bc^ | 4.31±0.01^bc^ | 4.15±0.21^c^ | 4.52±0.11^b^ | 5.78±0.04^a^ | 5.82±0.04^a^ | 5.86±0.03^a^ | 5.74±0.18^a^ | *** |
| 6 | 6.55±0.10^a^ | 4.99±0.08^c^ | 4.96±0.08^c^ | 5.02±0.08^c^ | 5.31±0.10^b^ | 4.99±0.12^c^ | 4.97±0.15^c^ | 5.02±0.13^c^ | 5.01±0.15^c^ | *** |
| 9 | 7.21±0.26^a^ | 5.82±0.15^b^ | 5.61±0.21^bc^ | 5.82±0.18^b^ | 5.72±0.05^bc^ | 5.52±0.07^bc^ | 5.54±0.13^bc^ | 5.39±0.16^c^ | 5.52±0.07^bc^ | *** |
| 12 | 7.79±0.02^a^ | <1^c^ | <1^c^ | <1^c^ | <1^c^ | 5.46±0.19^b^ | 5.61±0.11^b^ | 5.67±0.08^b^ | 5.58±0.16^b^ | *** |
| 15 | 7.78±0.11^a^ | <1^c^ | <1^c^ | <1^c^ | <1^c^ | 5.76±0.06^b^ | 5.69±0.14^b^ | 5.82±0.19^b^ | 5.88±0.06^b^ | *** |

^a^ ATCC 7644 indicates *Listeria monocytogenes* ATCC 7644. Trial 1: *Listeria monocytogenes* alone, positive control; trial 2: *Listeria monocytogenes*+Enterocin BH04; trial 3: *Listeria monocytogenes*+Enterocin BH12; trial 4: *Listeria monocytogenes*+Enterocin BH84; trial 5: *Listeria monocytogenes*+Enterocin BH99; trial 6: *Listeria monocytogenes*+*Enterococcus faecium* BH04; trial 7: *Listeria monocytogenes*+*Enterococcus faecium* BH12; trial 8: *Listeria monocytogenes*+*Enterococcus faecium* BH84, trial 9: *Listeria monocytogenes*+*Enterococcus faecium* BH99. Sig.: Degree of statistical significance, ns: Not statistically significant, *: p<0.05, **: p<0.01, ***: p<0.001. Lowercase letters represent statistical differences in the same row.
